# An Overview of IgG4-Related Disease

**DOI:** 10.26502/aimr.0259

**Published:** 2026-09-10

**Authors:** Amy Phu, Lannhi Nguyen, Daniel Mashiach, Ryan Afrahim, Devendra K Agrawal

**Affiliations:** 1 Department of Translational Research, College of Osteopathic Medicine of the Pacific, Western University of Health Sciences, Pomona, California 91766 USA.

**Keywords:** Autoimmune hepatitis, Dacryoadenitis, Immunoglobulin g4-related disease, Parotitis, Sialadenitis, Submandibular swelling

## Abstract

IgG4-related disease is a chronic inflammatory condition that has been formally described and extensively researched only in the recent few decades, making it a relatively new clinical entity. Furthermore, IgG4- related disease has been highly underreported and misdiagnosed as other similarly presenting conditions. In this article, we discuss the variable presentation, diagnosis process and criteria of IgG4-related disease and Sjogren’s syndrome in consideration of the literature currently available. In IgG4- related disease patients, an accurate diagnosis is crucial to timely treatment and circumvention of serious adverse outcomes such as irreversible organ damage.

This critical review article provides an overview of IgG4-related disease with a primary focus on (i) current understanding of its pathophysiological mechanisms, (ii) most recognized clinical phenotypes, (iii) current diagnostic guidelines, and (iv) current treatments available.

## Introduction

IgG4-related disease is a rare immune mediated condition first defined as its own clinical entity in 2003, when extra-pancreatic manifestations were identified in patients with autoimmune pancreatitis [1]. It has been increasingly recognized since its initial description approximately two decades ago. The incidence of IgG4-related disease in the United States is estimated to be 1.20 per 100,000 person-years, like the incidence of related conditions such as ANCA-associated vasculitis and systemic sclerosis. However, it is noted that this is a conservative estimate, given that the incidence of IgG4- related disease appears to be growing significantly due to expanding awareness within the medical community, nearly doubling from 2015 to 2019. Multiple studies have found the average age of IgG4-RD patients to be in the sixth decade of life, affecting individuals from diverse racial and ethnic backgrounds [2,3]. Prior studies have reported predominantly male cohorts, with a few exceptions [2,4]. There are estimates that IgG4-related disease is associated with a 2.5-fold higher risk of death [2]. Given IgG4- related disease ‘s increasing incidence and its potential for morbidity and mortality in untreated disease, it is imperative that we continue to expand awareness and gain further insight into IgG4-RD to reduce time lapsed from onset to diagnosis and treatment.

## Methods:

A review of the literature was conducted encompassing data from February 2003 to October 2025, with a primary emphasis on case reports and original research articles. A literature search was performed in PubMed using keywords including “IgG4”, “IgG4-RD”, “IgG4- related disease”, “Dacryoadenitis”, “Submandibular swelling”, and “Parotitis”. Of 65 articles initially reviewed, 27 were selected for inclusion based on relevance, quality, and English language. The reference list of relevant articles was also reviewed.

### Pathophysiology

The histopathological profile of the fibroinflammatory response in IgG4- related disease primarily features B lymphocytes, IgG-positive and IgG4-positive plasma cells, T lymphocytes, and lymphoid follicle formation with germinal centers and antigen-presenting cells. This is suggestive of a local antigen driven immune response and development of acquired lymphoid tissue [5]. Specific antigens and immune factors in confirmed cases of IgG4-related disease have not yet been identified. Fibrosis and obliterative phlebitis are both non-specific histological findings associated with inflammation. Additionally, increases in serum immunoglobulins such as IgG4 are present in a variety of chronic inflammatory diseases [6]. These characteristics are substantially diminished after administration of steroid therapy and remission and are increased during periods of active disease [6].

Circulating follicular T helped cells and B cells have been identified as the two T cell subsets in IgG4- related disease. Circulating follicular T helper cells expressing programmed cell death protein 1 are expanded in IgG4- related disease and are directly correlated with disease severity and serum IgG4 concentrations [6]. Circulating follicular T helper cells also produce IL-4 which plays a role in class-switching of B cells to both IgG4 and IgE and oligoclonal expansion of IgG4-positive B cells [6].

Clonal expansion of plasmablasts and CD4-positive cytotoxic T lymphocytes suggests an antigen-driven fibroinflammatory response, potentially to local autoantigens [5,7,10]. At the time of writing, gaps in knowledge include the exact triggers of disease onset, the role of IgG4, and whether the T and B cells involved are responding to the same antigenic drive; while antigen-specific antibodies have been identified in other diseases such as myasthenia gravis and chronic inflammatory demyelinated polyradiculopathy, none have been yet identified for IgG4- related disease [5,7,10]. Lastly, irreversible organ damage is attributable to the irreversibility of fibrosis and the mass effect of fibrosis and active inflammation on adjacent tissues and organs. Up to 60% of patients present with exocrine or endocrine damage to the pancreas at the time of diagnosis, leading to risk of development of diabetes and exocrine pancreatic insufficiency [5,7,10]. Further research is necessary to determine the precise pathogenesis and molecular mechanisms of IgG4- related disease.

Furthermore, specific genetic markers that may contribute to the disease process have not yet been confirmed, but recent studies have identified certain genes that may potentially play a role in the development of this multifaceted disease. In addition to the hallmark histopathologic findings of tissue-infiltrating IgG4 plasma cells and significant tissue fibrosis in the context of an inflammatory response, patients commonly have allergy manifestations alongside elevations in IgG4 and IgE levels. This led to the hypothesis that a Th2 response may trigger disease onset [11,12]. Th2 cytokines, such as IL-4 and IL-13, promote IgG4 and IgE class-switching and induce fibrosis [13,14]. However, the exact mechanisms underlying the Th2 bias in IgG4- related disease remain unclear and warrant further investigation.

The hypothesized contribution of Th2 response to disease instigation is supported by a 2024 study that identified shared variants in IKZF1, which encodes the transcription factor IKAROS, and the E3 ubiquitin ligase UBR4 [15]. These shared variants were identified in three family members with IgG4- related disease, linking these mutations to abnormal T cell signaling [15]. The IKZF1 variant increased transcription of the FYN promoter in T cells, while the UBR4 variant impaired lysosomal degradation of CD45, together lowering the activation threshold and creating a positive feedback loop that made T cells hyperresponsive, even to low-avidity autoreactive signals. Elevated FYN was also found to promote Th2 polarization through stabilization of the JunB transcription factor [15]. These findings suggest a mechanistic explanation for the Th2-skewed inflammation, atopy, and autoimmunity characteristic of IgG4- related disease.

### Clinical Presentations of IgG4-Related Disease

IgG4-related disease commonly demonstrates an insidious progression in terms of clinical presentation, with signs and symptoms often present for months or even years prior to establishment of the diagnosis. IgG4-related disease continues to present a diagnostic challenge due to its highly variable, multisystemic presentation, with a median delay of 9.5 months in diagnosis from initial presentation [2]. Systemic inflammatory symptoms such as fever are uncommon, although weight loss of 5-15 kg can gradually occur over months. Fatigue, arthralgias, and other musculoskeletal symptoms such as enthesopathy, or inflammation at the site of a tendinous insertion into a bone, are common in many patients. However, frank arthritis is rare and suggests a different diagnosis [1]. Generally, the characteristic pathological features include lymphoplasmacytic infiltration with abundant IgG4 and B cells, in addition to fibrosis in the affected tissues, arranged in a storiform pattern [2,11]. Spontaneous remission of IgG4-related disease in the absence of any treatment has also been reported, further complicating the diagnostic picture [9].

Any solid organ or anatomic site may be affected in IgG4-related disease; however, there are four main clinical phenotypes that have been identified based on organ involvement: head and neck-limited disease, pancreato-hepato-biliary disease, retroperitoneal fibrosis and/or aortitis, and classic Mikulicz syndrome with systemic involvement (figure 1) [1]. These phenotypes were identified through latent class analysis of 765 cases from international cohorts and subsequently validated in a replication cohort. Group 1 (Pancreato-Hepato-Biliary disease) comprises approximately 31% of cases, Group 2 (Retroperitoneal Fibrosis and/or Aortitis) accounts for 24%, Group 3 (Head and Neck-Limited disease) represents 24%, and Group 4 (classic Mikulicz syndrome with systemic involvement) comprises 22% of patients [1]. Each phenotype demonstrates dominant immune cell subsets unique to its pathogenesis [7].

Additionally, each distinctive phenotype has been found to differ from one another demographically, providing a potential framework for recognition and diagnosis. One large cohort study of both Asian and non-Asian countries suggested that cases of Asian descent are particularly predisposed to developing IgG4-related disease complications limited to the head and neck region, while predominantly Caucasians have an increased predilection for pancreato-hepatobiliary disease and/or retroperitoneal and aorta disease [1].

Pancreato-hepato-biliary disease most commonly presents with obstructive jaundice secondary to pancreatic swelling and bile duct compression. Patients may develop endocrine or exocrine pancreatic failure, leading to diabetes mellitus, malabsorption, and weight loss. Autoimmune pancreatitis is frequently associated with IgG4-related sclerosing cholangitis affecting both intrahepatic and extrahepatic bile ducts [8]. Retroperitoneal fibrosis and/or aortitis target three anatomical locations: periaortic/arterial regions, periureteral areas, and plaque-like masses broadly involving the retroperitoneum. IgG4-related aortitis can cause thoracic aortic aneurysms or dissections, though unlike giant cell and Takayasu’s arteritis, it tends to spare primary aortic branches. Head and neck-limited disease most commonly affects females and Asian patients. Common manifestations include allergic rhinitis, nasal polyps, chronic sinusitis, and nasal obstruction. Lacrimal gland enlargement is the most common ophthalmic feature, and the classic triad of dacryoadenitis with parotid and submandibular gland enlargement (Mikulicz disease) has a high positive predictive value for IgG4-related disease [1,8]. Classic Mikulicz syndrome with systemic involvement has been observed to demonstrate the highest median serum IgG4 concentration (1170 mg/dL) and presents with salivary and lacrimal gland swelling in addition to multiorgan involvement [1].

Although clinician awareness of these four subtypes may certainly aid in timely disease recognition and management, IgG4-related disease remains kaleidoscopic in its clinical manifestations, continuing to pose a significantly complex diagnostic challenge. One case describes a patient with overlapping features of both the head and neck-limited phenotype with the Mikulicz/systemic phenotype; IgG4-related disease diagnosis was only able to be confirmed through positron emission tomography-guided lung biopsy with specialized immunohistochemical staining [3].

### Diagnosis of IgG4-Related Disease

Diagnosis of IgG4-related disease is established when the following criteria are met (Figure 2): clinical/radiologic evidence of tumor-like swelling of an involved organ, and a biopsy demonstrating lymphoplasmacytic infiltrate enriched in IgG4-positive plasma cells, storiform fibrosis, and obliterative phlebitis. Biopsies of the lacrimal mass, labial salivary glands, and submandibular glands have all been reported in the literature. Biopsy of swollen glands, particularly of the submandibular and parotid glands, is crucial to establishing a definitive diagnosis, as other clinical entities such as marginal zone B cell lymphoma and SS may present as mimickers [6]. Minor salivary gland biopsies are an effective and less invasive measure of diagnosing IgG4-related disease, as there have been reports of such samples showing significant IgG4 positive plasma infiltration [7].

The classification criteria for IgG4-related disease adopted a value of 135 mg/dL as the cut-off value [3]. However, a 2015 study noted that elevated serum IgG4 concentrations had only 60% specificity and a 34% positive predictive value. The authors also noted doubling the cutoff for IgG4 improved specificity (91%) but decreased sensitivity (35%) [14]. Elevated serum IgG4 levels alone are insufficient for diagnosis, and serum IgG4 levels may be normal even in confirmed cases of disease. Thus, confirmation via tissue biopsy is critical to decrease the likelihood of misdiagnosis and unnecessary immunosuppressive therapy.

Although parotid gland swelling is a relatively uncommon phenomenon compared to submandibular swelling and dacryoadenitis in reported IgG4-related disease cases, IgG4-related disease should still be considered within the differentials for patients presenting with parotid gland swelling alongside other clinical and pathologic considerations including major salivary and lacrimal gland enlargement, orbital disease, autoimmune pancreatitis, retroperitoneal fibrosis and tubulointerstitial nephritis. Notably, fever is rare while lymphadenopathy is common. Asthma or allergy are found in approximately 40% of patients [17].

Biopsy in suspected IgG4-related disease patients may show storiform fibrosis, although this finding is not required for definitive diagnosis. Biopsy of the submandibular gland is most sensitive for establishing the diagnosis for IgG4-related disease, although labial salivary gland biopsy may also be used but with greater caution due to lesser sensitivity [16].

### Treatment

Treatment of IgG4-related disease is urgently required in most cases, particularly in symptomatic patients presenting with multi-organ involvement. Early recognition and treatment are important to reduce inflammation, prevent organ damage, and severe fibrotic disease [1]. Additionally, early treatment can help progression from treatment-responsive to non-responsiveness in the disease course. Importantly, some asymptomatic patients may also warrant treatment, as the indolent nature of the disease may lead to severe and irreversible damage to the biliary tree, kidney, aorta, retroperitoneum, and other organs [18]. These considerations highlight the need for a proactive and individualized approach to treatment.

Glucocorticoids remain the first-line treatment for IgG4-related disease. Initial therapy is prednisone monotherapy, typically at a dose of 0.6 mg/kg once daily. Patients will typically demonstrate improvements in symptoms, laboratory markers, and organ involvement within two to four weeks. After a clinically significant response is evident in the affected organ system, glucocorticoids are tapered gradually over 2 months to achieve the end goal of complete discontinuation of the medication. At the time of writing, it is still unclear whether patients with IgG4-related disease should routinely receive remission maintenance therapies such as rituximab. However, recent studies imply that IgG4-related disease has a substantial relapse rate, which may be reduced through maintenance therapies. Roughly 10-53% of patients fail to enter remission or relapse within a year of initial treatment. Risk factors for relapse include multiorgan disease, elevated IgG4 levels, IgE, and circulating eosinophils [2].

Due to the risk of subclinical progression, patients require close monitoring with laboratory values, specifically serum IgG4 levels, changes in symptoms, and serial imaging. One study revealed that waiting to initiate treatment with mild symptoms and low disease activity has a higher relapse rate than the subgroup that received treatment immediately [3].

Given the high relapse rates associated with glucocorticoid monotherapy, steroid-sparing immunosuppressive agents are frequently used for maintenance or relapse therapy. Among these, rituximab has emerged as a highly effective option, particularly for patients with refractory, relapsing, or organ-threatening IgG4-related disease. By targeting CD20-positive B cells, rituximab, a chimeric antibody, induces depletion of pathogenic B-cell populations and reduces disease activity, demonstrating efficacy in inducing remission in patients who fail to respond to glucocorticoids or conventional immunosuppressants [4,8]. Additionally, combining rituximab with initial glucocorticoid therapy has been associated with lower relapse rates compared to glucocorticoid monotherapy alone [4].

New generation B-cell therapies such as humanized monoclonal antibody inebilizumab are expanding treatment options. Inebilizumab, an anti-CD19 monoclonal antibody, offers broader depletion of B-cell lineages, including plasmablasts, and has shown promising results in clinical trials (5). Patients treated with inebilizumab had lower rates of relapse and were more likely to achieve flare-free, glucocorticoid-free complete remission [6].

Furthermore, CD19-directed chimeric antigen receptor (CAR) T-cell therapy represents a novel treatment option for severe, treatment-refractory IgG4-RD. By inducing an “immune reset” through eliminating nearly all CD19+ B cells, allowing the body to rebuild a new B-cell population, these patients were able to either achieve drug-free remission or had major improvement of multiorgan involvement [7].

Other steroid-sparing immunosuppressive agents include azathioprine, mycophenolate mofetil, and methotrexate may be used as maintenance therapy for relapsing disease. Azathioprine has been utilized as a steroid-sparing maintenance alternative, demonstrating a potential role in maintaining remission and reducing glucocorticoid dependence. However, due to the variable responses and a significant proportion of patients experiencing relapse or requiring treatment escalation to monoclonal therapies, azathioprine treatment may be less effective than B-cell targeted therapies [9]. Similarly, combination mycophenolate and glucocorticoid have a higher efficacy than treatment with glucocorticoid monotherapy [10].

One case report demonstrates successful treatment of IgG4-related dacryoadenitis with JAK inhibitor baricintinib [11]. This reveals how medications targeting JAK-STAT family may be effective in patients who failed monoclonal treatments and may need an alternative treatment pathway.

These case reports reveal the intricacies and complexities of treating IgG4-related disease. The overall management of IgG4-related disease is highly individualized and mechanism-driven. Though glucocorticoids remain the first line therapy, the high rates of relapse suggest that early integration of steroid-sparing and biologic therapies may help reduce relapse and achieve drug-free remission. Larger randomized trials and comparisons between the efficacies of therapies may help continue to guide treatment and improve long-term outcomes for IgG4-related disease.

## Conclusion

IgG4-related disease remains a profoundly complex clinical entity characterized by its highly variable, multisystemic presentation that frequently mimics other inflammatory conditions. Early and accurate identification is imperative to circumvent severe, irreversible organ damage through timely intervention. As recognition of IgG4-related disease expands, continued research into its precise Th2-driven pathophysiology, non-invasive biomarkers, and advanced cellular therapies will be crucial for refining diagnostic criteria and improving patient outcomes.

## Figures and Tables

**Figure 1:**
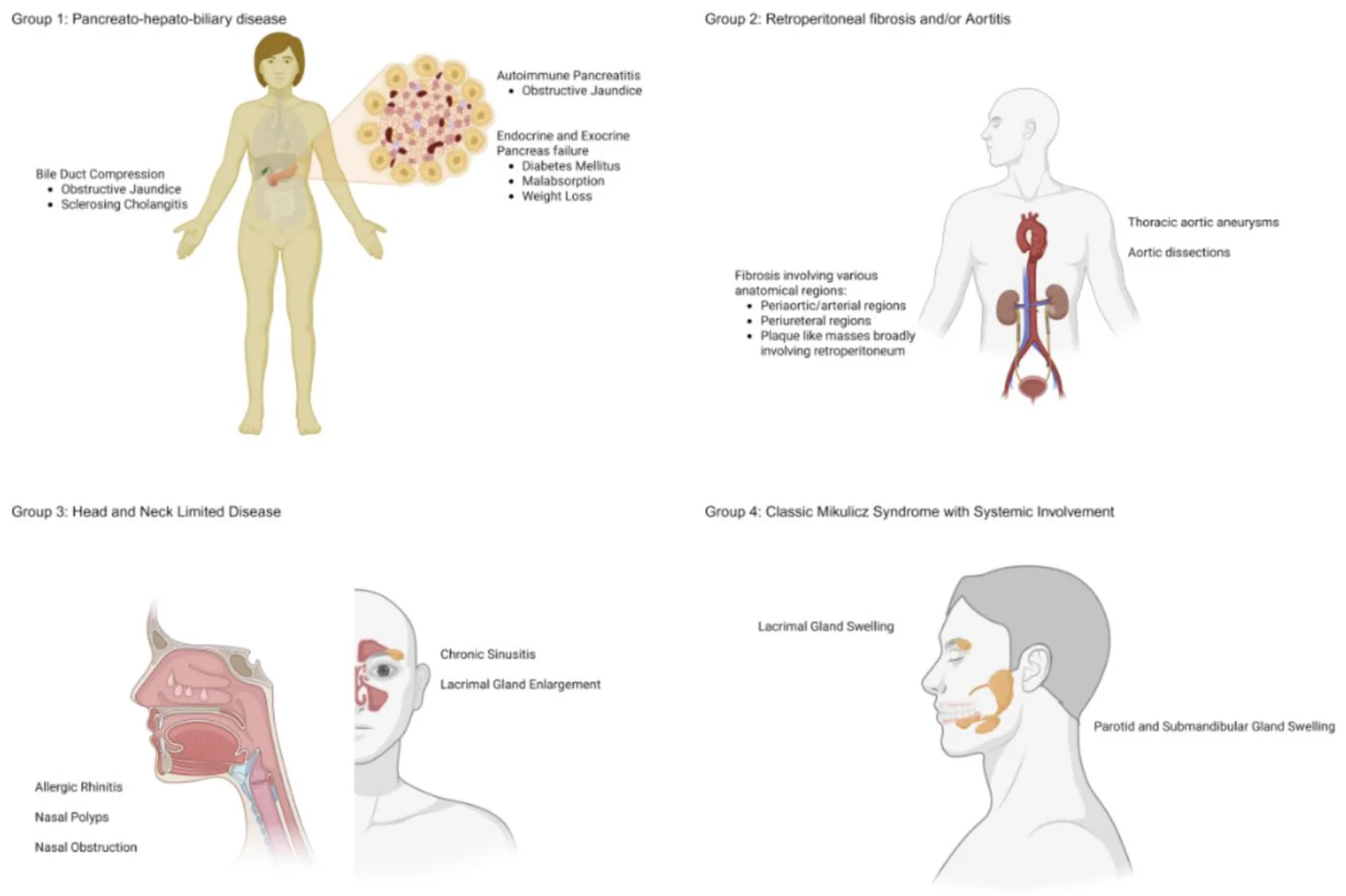
The four major phenotypes of IgG4-related disease.

**Table 1:** Summary of the 2020 Revised Comprehensive Diagnostic (RCD) Criteria for IgG4-Related Diseases.

Detailed Diagnostic (RCD) Criteria for IgG4-Related Diseases	
1. Clinical and radiological feature	One or more organs show diffuse or localized swelling, mass, nodule characteristic for IgG4-related disease. In single organ involvement, lymph node swelling is omitted.
2. Serological diagnosis	Serum IgG4 levels are more than 135 mg/dL.
3. Pathological diagnosis	Positivity for two of the following three criteria:
	a. Dense lymphocyte and plasma cell infiltration with fibrosis.
	b. Ratio of IgG4-positive plasma cells/IgG-positive cells greater than 40% and the number of IgG4-positive plasma cells greater than 10 per high-powered field
	c. Typical tissue fibrosis, particularly storiform fibrosis, or obliterative phlebitis*
Diagnostic Classification	**Definite**: if +ve clinical and radiological features, +ve serological diagnosis, and +ve pathological diagnosis
	**Probable**: if +ve clinical and radiological features and +ve pathological diagnosis
	**Possible**: if +ve clinical and radiological features and +ve serological diagnosis

## Key Points:

- IgG4-related disease incidence is growing significantly due to expanded medical awareness, typically presenting in the sixth decade of life and carrying a potential 2.5-fold higher risk of death if untreated.
- The disease process is driven by an antigen-driven fibroinflammatory response and Th2-skewed inflammation, which recent studies suggest may be mechanistically linked to shared variants in the IKZF1 and UBR4 genes.
- Clinical presentations can be categorized into four major phenotypes: pancreato-hepato-biliary disease, retroperitoneal fibrosis and/or aortitis, head and neck-limited disease, and classic Mikulicz syndrome with systemic involvement.
- Elevated serum IgG4 levels (cutoff of 135 mg/dL) are insufficient for a standalone diagnosis due to low specificity, making histopathological confirmation strictly necessary to avoid unnecessary immunosuppressive therapy.
- While labial salivary gland biopsies are less invasive, submandibular gland biopsy is the most sensitive and reliable method for establishing a definitive diagnosis.
- Although glucocorticoids are the standard initial treatment, a 10-53% relapse rate frequently necessitates the integration of therapies like rituximab, the anti-CD19 monoclonal antibody inebilizumab, or CAR T-cell therapy to achieve drug-free remission.
